# Surgical Treatment in a High-Risk Pulmonary Embolism: Case Report

**DOI:** 10.3390/medicina57070725

**Published:** 2021-07-17

**Authors:** Horatiu Moldovan, Andra-Madalina Sibisan, Robert Tiganasu, Elena Nechifor, Daniela Gheorghita, Ondin Zaharia, Mihai Albu, Daniela Popescu, Adrian Molnar, Mihaela Craciun, Alexandru Scafa

**Affiliations:** 1 Faculty of Medicine, Carol Davila University of Medicine and Pharmacy, 014461 Bucharest, Romania; h_moldovan@hotmail.com (H.M.); ondin.zaharia@gmail.com (O.Z.); alexscafa@yahoo.com (A.S.); 2 Emergency Clinical Hospital Bucharest, 014461 Bucharest, Romania; andrasibisan@yahoo.com (A.-M.S.); tiganasu.robert@yahoo.com (R.T.); 3 SANADOR Clinical Hospital, 010991 Bucharest, Romania; nechiforelena21@yahoo.com (E.N.); dani31popescu@yahoo.com (D.P.); mihaela.craciun@sanador.ro (M.C.); 4 Faculty of Materials Science and Engineering, Politehnica University of Bucharest, 060042 Bucharest, Romania; 5 Prof. Dr. Theodor Burghele Clinical Hospital, 050659 Bucharest, Romania; 6 Clinical Hospital Ploiesti, 100337 Ploiesti, Romania; dralbu@yahoo.com; 7 Iuliu Hateganu University of Medicine and Pharmacy, 400012 Cluj Napoca, Romania; adrianmolnar097@gmail.com; 8 Heart Institute, 400001 Cluj Napoca, Romania

**Keywords:** major pulmonary embolism, emergency pulmonary embolectomy, modified trendelenburg procedure

## Abstract

We present the case of a 35-year-old woman who had a high-risk pulmonary embolism (according to ESC risk stratification for pulmonary embolism) after she had undergone a Caesarion section. Postoperatively, she presented with acute left lower limb pain, swelling and erythema. A diagnosis was made of deep vein thrombosis (DVT) of the ilio-femoral and popliteal veins. She was started on anticoagulant therapy, which proved to be inefficient, the patient developing a left calf and thigh oedema and shortness of breath. A CT scan revealed high-risk embolus located in the right atrium and through the tricuspid valve. The decision was made to refer her to a cardiovascular surgeon. During her preoperative evaluation, the patient became hemodynamically unstable and was rushed into the operating room, severely desaturated, bradycardic, without consciousness, with severe hypotension. On the basis of the severe state of the patient and the CT scan findings we performed an emergency pulmonary embolectomy, with the patient on cardio-pulmonary by-pass, without cross-clamping the aorta, using a modified Trendelenburg procedure. This case supports using open pulmonary embolectomy for patients with hemodynamic instability on the basis of clinical diagnosis.

## 1. Introduction

Aggressive emergency treatment is necessary to decrease the high mortality rates of the high-risk pulmonary embolism (PE) [1]. There is as strong debate concerning the appropriate course of treatment for pulmonary embolism, many algorithms and guidelines have been made, but there are few prospective studies [2]. Treatment plans include thrombolysis, catheter embolectomy and surgical embolectomy [3,4]. This is the case report of a 35-year-old female presenting with high-risk PE three weeks after a caesarean section, following a DVT, that underwent a surgical trombembolectomy via a modified Trendelenburg operation. The aim of the current case report is to demonstrate the significance of the surgical approach in a high-risk pulmonary embolus. Even though the less invasive means of treatment of PE have gained more ground over the last 20 years, the importance of the surgical approach in selected cases must not be dismissed [5].

## 2. Case Report

**Figure 1:**
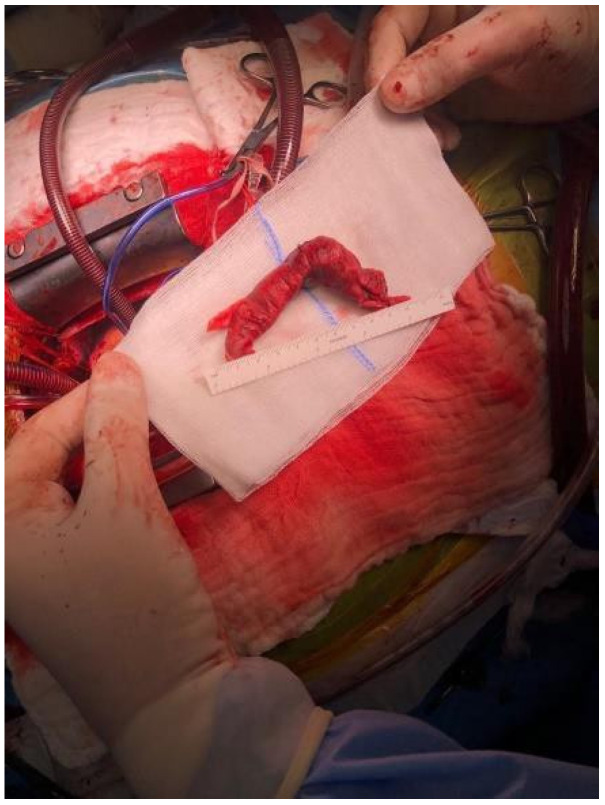
Massive thrombus from the main pulmonary artery.

**Figure 2:**
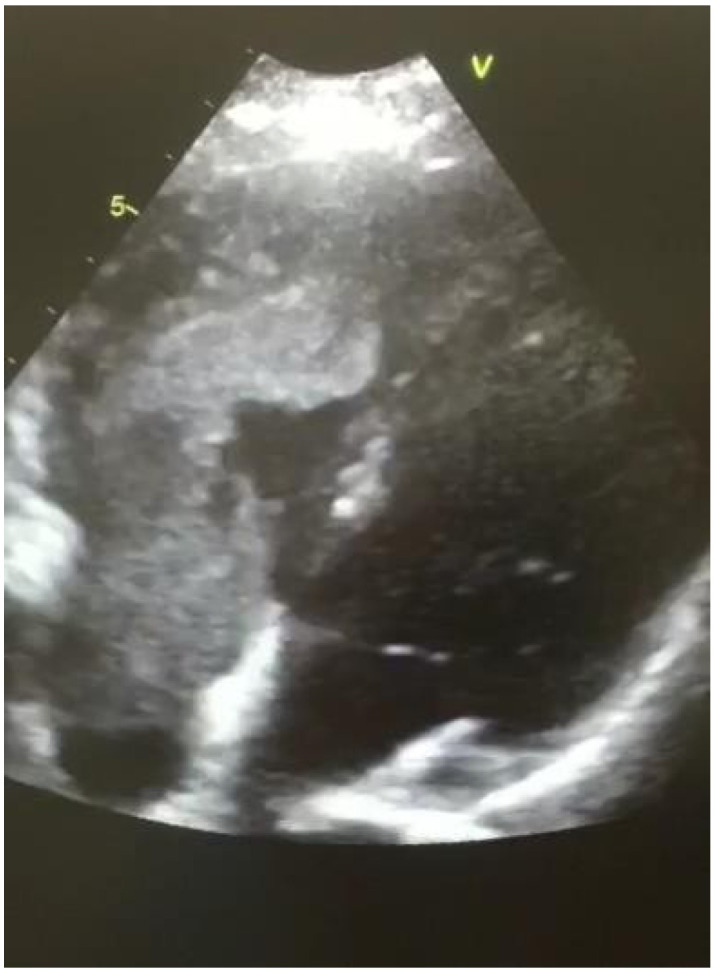
Image of transthoracic echocardiography showing the presence of a massive embolus in the right atrium and through the tricuspid valve.

We present the case of a 35-year-old female admitted to the hospital after a cesarian section who presented with acute left lower limb pain, swelling and erythema. A Doppler compression sonogram test revealed proximal DVT of the ilio-femoral and popliteal veins. Anticoagulant therapy was immediately initiated with unfractionated heparin. The treatment proved inefficient, the patient developed a massive left calf and thigh oedema and shortness of breath. Contrary to her clinical state, the troponin and BNP levels were in the normal range (troponin I—1.9 pg/mL and BNP—11.1 pg/mL). The D-dimer levels were high—13.332 ng/mL and the CT scan revealed that the deep venous thrombosis extended to the inferior vena cava and a massive thrombus was situated in the right atrium and through the tricuspid valve. Figure 1 presents the massive thrombus after removal. In Figure 2 a transthoracic echocardiography showing the presence of a massive embolus in the right atrium and through the tricuspid valve is presented. Taking into consideration the size and location of such a thrombus, the patient was referred to a cardiovascular surgeon. During the preoperative evaluation of the patient further exams revealed a before unknown inherited thrombophilia, which revealed positive results for the mutations of the EPCR G4678C gene (Homozygous), prothrombin G20210A gene (Heterozygous), Factor XIII mutation V34L (Heterozygous), MTHFR C6677T (Heterozygous) and PAI1 4G/5G (Heterozygous), the X-ray was clean, the transthoracic echocardiography showed dilated right cavities, an 8-cm long thrombus passing through the tricuspid valve, a thrombus in the inferior vena cava, dilated pulmonary artery and a PVAT (pulmonary velocity acceleration time) value of 43 ms, which corelates to a severely elevated PAP. No risk factors for venous thromboembolism, neither acquired nor congenital were confirmed during her preoperative exams, other than her inherited thrombophilia.

The patient became hemodynamically unstable and was rushed into the operating room, severely desaturated, bradycardic, without consciousness, with severe hypotension. After intubation and general anesthesia, a transesophageal echocardiography was performed and showed clear right cavities and complete obstruction of the main pulmonary artery and the right branch with a giant thrombus. A direct thrombectomy was performed through median sternotomy, cardiopulmonary bypass without aortic cross clamp, with bicaval cannulation. This approach was elected for maximum visibility given the hemodynamical instability of the patient.

Postoperatively, the patient was extubated after only 4 h, and was weaned of the vasopressor support on the first day. She was discharged after one week with persistent left calf and thigh oedema for which she was prescribed warfarin and beta-blockers. After two weeks, the patient was readmitted in the hospital for a scheduled interventional thrombaspiration of the inferior vena cava and balloon dilatation of the left common iliac vein and common femoral vein with good angiographic results. The postoperative Doppler check-up showed left superficial femoral vein compressible by 80%, with residual thrombus (0.46 cm in diameter), left common femoral vein with permeabilization flow, with residual thrombus and the left popliteal vein completely compressible. The transthoracic echocardiography revealed adequate cardiac function.

## 3. Discussion

This case report details the natural history of a high-risk pulmonary embolism. Acute high-risk pulmonary embolism leads to a rapid rise in pulmonary vascular resistance which in turn compromises the right ventricular contractile function with the onset of right ventricular failure [6]. For patients with acute high-risk pulmonary embolism, there is a small window for obtaining a definitive diagnosis. Ideally, the diagnosis of pulmonary embolism can be obtained from the patient history and physical examination along with some selective tests, for example, an electrocardiography to rule out a myocardial infarction [7]. Signs of pulmonary embolism on the electrocardiography can be seen in approximately 50% of the cases (S1Q3T3 pattern, newly discovered right bundle branch block) [8]. Spiral CT pulmonary angiography is the gold standard in the diagnosis of acute pulmonary embolism [9], investigation that could cost valuable time in the case of a hemodynamically unstable patient. Transesophageal echocardiography is a fast, non-invasive method of diagnosing acute pulmonary embolism, being able to evaluate the right ventricular dilatation and the presence of emboli within the pulmonary arteries [10]. Precise recognition of possible complications is important for the medical and surgical management of these patients, as those complications could generate unexplained congestive heart failure and hemodynamic deterioration [11]. The importance of involving multidisciplinary teams (including specialists in cardiology, cardiac surgery, and intensive care) in complex cases with high risks is revealed by the successful outcome [12,13,14,15,16]. After the diagnosis of a massive pulmonary embolism is made, there must be immediate initiation of medical or surgical treatment. In a hemodynamically unstable patient, the decision to perform surgical embolectomy can be made on clinical criteria [17].

The types of treatment in acute pulmonary embolism vary with the clinical presentation. Anticoagulation and thrombolysis are the standard means of treatment for acute pulmonary embolism, but these are to be reserved for hemodynamically stable patients [18]. Clinical data suggest that patients being treated by thrombolysis are at a higher risk of death by increased risk of major hemorrhage and a high probability of pulmonary embolism recurrence compared with patients treated by pulmonary embolectomy [19,20].

Performing emergency surgical embolectomy or catheter embolectomy with fragmentation is recommended for patients with high-risk or intermediate high-risk pulmonary embolism with right ventricle dysfunction, contraindications to fibrinolysis or failed thrombolysis, in centers with the appropriate expertise and resources [21]. Very important to determining the best therapeutic strategy are the individual center and practitioner’s experience.

Alternatives that reduce the hemodynamic implications of pulmonary embolism without the burden of the thrombolysis or the cardiopulmonary bypass are catheter-based techniques. These techniques often use both mechanical methods (such as balloon angioplasty, mechanical fragmentation and suction thrombectomies) and pharmacological means (delivering a high concentration of thrombolytic agents at the site of obstruction) for thrombus reduction, thus reducing necessary dosage of thrombolytic agent and increasing efficacy with a lower risk of hemorrhage [22].

Clearance of the embolic burden may be better managed through surgical embolectomy, especially in cases with central pulmonary embolism and a larger embolic burden. On the other hand, in patients with contraindications for cardiac surgery and patients with less surgically accessible embolic disease, catheter assisted embolectomy may be the treatment of choice. Patients with high-risk pulmonary embolism may require artificial right ventricular and/or lung mechanical support. ECMO and other techniques are easier to undertake in the operating theater, and in most large-volume medical centers cardiac surgeons have the most experience with these techniques in safer conditions [23].

Our patient was initially treated for her DVT using anticoagulants (unfractionated heparin), which was ineffective. The fast decline of the patient, the high mortality risk of this particular thrombus and the relative contraindication for thrombolytic treatment due to her recent history of surgical intervention [24] limited the choice in the course of treatment to emergency surgical embolectomy [25]. We believe that surgical embolectomy is an adequate treatment for patients with hemodynamic instability, after ruling out other causes of hemodynamic collapse [26]. A possibility that was left unexplored due to the rapid decrease of the patient’s status was the off-pump surgical embolectomy, an approach that could have decreased the oxidative stress, inflammatory response and morbidity [27]. Additional studies should be carried out to establish the use of off-pump procedure. Furthermore, a minimally invasive approach should be considered in selected cases to reduce the risks associated with reintervention through median sternotomy thus decreasing the postoperative morbidity [28].

## 4. Conclusions

The choice to approach this patient surgically was the best course of treatment for this case of massive pulmonary embolism. Furthermore, it was the only therapeutic intervention that had a significant positive impact on the patient’s quickly worsening state.

Other minimally invasive techniques have been considered for the treatment of our patient, such as catheter-based techniques, with a possible immediate relief of some of the hemodynamic status of the patient. The surgical approach was the best choice of treatment, taking into account the better clearance of the embolic burden in our case of central pulmonary embolism, the surgical embolectomy being conducted under direct vision, having the ability to extract a central clot, clots extending into lobar vessels and even segmental branch vessels.

This case report is as an appropriate example of the utility of surgical embolectomy in massive pulmonary embolism, and it is our hope that it makes the case for earlier initiation of surgical treatment in such intricate cases.

## Data Availability

The study did not report any data.
